# Sepsis in Patients Hospitalized in Sicily, Italy, over the Period of 2016–2020: A Retrospective Study

**DOI:** 10.3390/jcm13082239

**Published:** 2024-04-12

**Authors:** Luca Pipitò, Romano Puccio, Domenico Marrali, Alessandro Mancuso, Maria Chiara Gagliano, Roberta Gaudiano, Manfredi Piccione, Chiara Iaria, Antonio Cascio

**Affiliations:** 1 Department of Health Promotion, Mother and Child Care, Internal Medicine and Medical Specialties “G D’Alessandro”, University of Palermo, 90133 Palermo, Italy; luca.pipito@community.unipa.it (L.P.);; 2 Infectious and Tropical Disease Unit, Sicilian Regional Reference Center for the Fight against AIDS, AOU Policlinico “P. Giaccone”, 90127 Palermo, Italy; 3 Infectious Diseases Unit, ARNAS Civico-Di Cristina-Benfratelli Hospital, 90127 Palermo, Italy

**Keywords:** sepsis, septic shock, hospitalization features, mortality, sepsis mortality, septic shock mortality, Italian sepsis, Italy

## Abstract

**Background:** Epidemiological data regarding the incidence and mortality of sepsis are scarcely available. The present study aimed to delineate the epidemiology of sepsis and related mortality among patients hospitalized in Sicily, Italy. **Methods:** Data on the discharge forms of all patients with sepsis in all Sicilian hospitals from January 2016 to December 2020 were retrospectively collected. **Results:** In Sicily, 15,373 hospitalizations associated with sepsis occurred from 2016 to 2020, with an overall in-hospital mortality rate of 36.3%. The percentage of hospitalizations associated with sepsis represented 0.65% of all admissions, with an increase over the years. Most patients were admitted to non-surgical and non-infectious disease units, accounting for 50.3% of total admissions. Stratification of patients according to age, intensive care unit (ICU) admission, and presence of septic shock revealed variations in in-hospital mortality rates. Among adult ICU-admitted patients with septic shock, mortality was highest at 81.0%, followed by non-ICU adult patients with septic shock (63.5%), ICU pediatric patients with septic shock (56.7%), ICU adult patients without septic shock (43.9%), non-ICU pediatric patients with septic shock (37.9%), non-ICU adult patients without septic shock (17.9%), ICU pediatric patients without septic shock (10.4%), and non-ICU pediatric patients without septic shock (1%).

## 1. Introduction

Sepsis is a severe and life-threatening disease characterized by organ dysfunction; it is caused by a dysregulated host response to an infection that, without prompt intervention, often progresses to septic shock, leading to a higher risk of mortality. Previous definitions distinguished sepsis, severe sepsis, and septic shock. In the Sepsis-3 Consensus, the definition of severe sepsis was abolished, and the presence of organ dysfunction was considered a necessary condition to diagnose sepsis [1]. Sepsis is a leading cause of in-hospital mortality and morbidity globally [1,2,3]. Administrative databases are increasingly used in research studies to capture clinical outcomes such as sepsis. Previous analyses based on explicit and implicit sepsis-related data according to International Classification of Diseases (ICD) codes showed the burden and mortality associated with sepsis [4,5,6,7]. The ICD codes for implicit sepsis specify an association between an infection and organ dysfunction. Clinical studies have also highlighted features of in-hospital-acquired and community-acquired sepsis [8,9,10]. Their results show that sepsis is a major public health concern, and the burden is high, particularly in intensive care units (ICUs). However, only a few studies dealt with non-ICU hospitalizations associated with sepsis. Individuals with chronic medical conditions, such as diabetes and chronic kidney disease, are at increased risk of sepsis events [11]. In 2017, an estimated 48.9 million incident cases of sepsis were recorded worldwide, and 11 million sepsis-related deaths were reported, representing 19.7% of all global deaths [4]. In Italy, a few studies have investigated the epidemiology and features of hospitalizations associated with sepsis [12,13,14,15]. Sicily is the largest region in Italy and the fifth most populous region, with a population of 4,801,468 inhabitants (8.1% of the total 58,983,122 Italian inhabitants). Hospitalizations in Sicily represent approximately 7% of national hospitalizations. The objective of this study is to delineate the epidemiology of sepsis and related in-hospital mortality among patients hospitalized in Sicily.

## 2. Materials and Methods

A retrospective study was conducted to examine all hospitalizations associated with sepsis in Sicily. Data on the discharge forms of all patients with sepsis in all public and private Sicilian hospitals from January 2016 to December 2020 were retrospectively collected from hospital standard discharge forms (H-SDFs), and the subsequently analyzed H-SDF data were compiled by clinicians after patient discharge or patient death. This study included all individuals with an International Classification of Diseases, Ninth Revision (ICD-9) code indicative of a diagnosis of sepsis, septicemia, or septic shock as recorded in the H-SDFs. In the H-SDFs, up to six diagnoses could be reported, and all of them were checked.

The ICD-9 is a coding system used to classify and code diagnoses and procedures in healthcare settings. It provides a standardized method for capturing and reporting medical information, thus facilitating data collection, analysis, and reimbursement processes. The ICD-9 system consists of alphanumeric codes that represent various diseases, conditions, and medical procedures.

We considered only explicit diagnosis of sepsis according to the ICD-9 codes, as in a previous work [16]:

- Septic shock is specified by ICD-9 code 785.52;
- Severe sepsis without shock is specified by ICD-9 code 995.92;
- Non-severe organism-specific sepsis, including septicemia, is specified by organism-specific sepsis codes (ICD-9 codes: 0380, 03810, 03811, 03812, 03819, 0382, 03840, 03841, 03842, 03843, 03844, 03849, 0388, 0031, 0362, and 0545);
- Neonatal septicemia is specified by ICD-9 code 771.81;
- Non-severe sepsis, including unspecified septicemia, is specified by ICD-9 code 0389.

For our data analysis, we did not differentiate between sepsis and severe sepsis, in alignment with the Sepsis-3 Consensus. Instead, we solely distinguished between sepsis and septic shock. The following variables were assessed for each patient: age, sex, length of stay (LOS), charges, death, main comorbidities, and pathogens and associated infections. Admission costs were derived from reimbursements made by the Italian Health System to hospitals based on each diagnosis-related group. As this computerized system is anonymous, according to the Italian Data Protection Authority, neither ethical committee approval nor informed consent was required. Regional health authorities routinely use anonymous data for epidemiological and administrative purposes. The patients were subdivided according to age (≤16 vs. >16 years) and ICU admission. Demographic characteristics, comorbidities, and coinfections available on the H-SDFs were analyzed.

### Statistical Analysis

Continuous variables are summarized as mean ± standard deviation, whereas categorical variables are presented as absolute and relative frequencies. Annual trends for the number of sepsis cases were investigated using a linear regression model with estimates of unstandardized coefficients (ß) and their confidence intervals (95% CI). Differences in means were evaluated using an unpaired Student’s *t*-test, and the χ^2^ test was applied to categorical variables. Statistical significance was set at a *p*-value of <0.05. Crude odds ratios (ORs) and their 95% CI for the association between two variables were calculated. The IBM SPSS Statistics software (Version 26) was used to conduct the statistical analysis, and Sankeymatic was used to construct a Sankey plot.

## 3. Results

**Figure 1:**
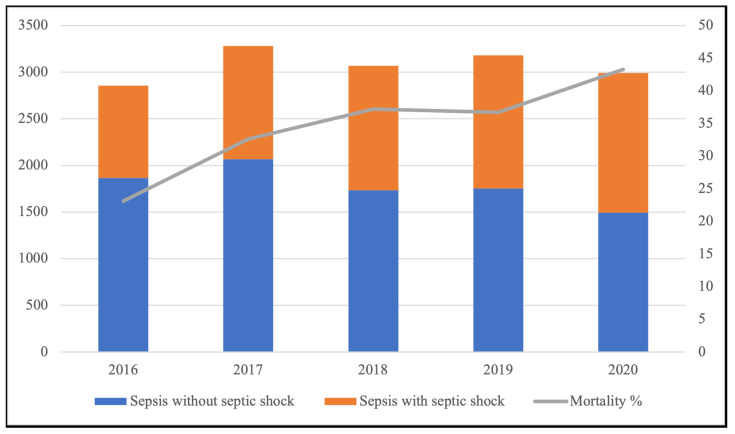
Trend of hospitalization associated with sepsis and in-hospital mortality over the years in Sicily from 2016 to 2020.

In Sicily, a total of 15,373 hospitalizations associated with sepsis occurred from 2016 to 2020. The number of deaths was 5580, and the in-hospital mortality rate increased from 23.1% to 43.0% (*p* = 0.061) (Figure 1).

**Figure 2:**
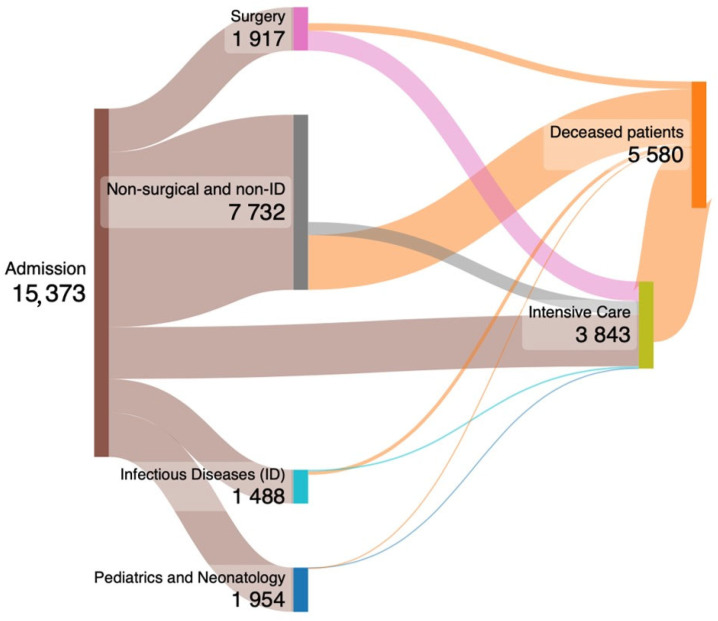
This figure illustrates the admission process, transfers to ICUs, and outcomes of patients who developed sepsis. Among them, 2282 patients were directly admitted to ICUs, while 1561 were transferred from other departments during their hospitalization. In-hospital mortality rates varied by admission department: 61.1% for surgical units, 52.4% for ICUs, 14.8% for infectious disease (ID) units, 38.4% for non-surgical and non-ID units, and 1.3% for pediatric and neonatology units.

The average percentage of hospitalizations associated with sepsis compared with the number of hospitalizations for all causes (n = 2,353,271) in Sicily was 0.65%, and the range was from 0.57% to 0.76% between 2016 and 2020, with an increase over the years (ß = 0.892; 95% CI: 0.00285, 0.07515; *p* = 0.041). The hospitalization rate per 100,000 Sicily inhabitants presented an average of 62.2 ± 3.6. The majority of patients were admitted to non-surgical and non-infectious disease units, accounting for 50.3% of total admissions. A smaller proportion of patients (14.8%) were admitted to ICUs, while 12.7% were admitted to pediatric and neonatology units, 12.5% to surgery units, and 9.7% to infectious disease units (Figure 2). Admissions, transfers of patients to ICUs after admission, and outcomes of patients are depicted in Figure 2 using a Sankey plot.

**Figure 3:**
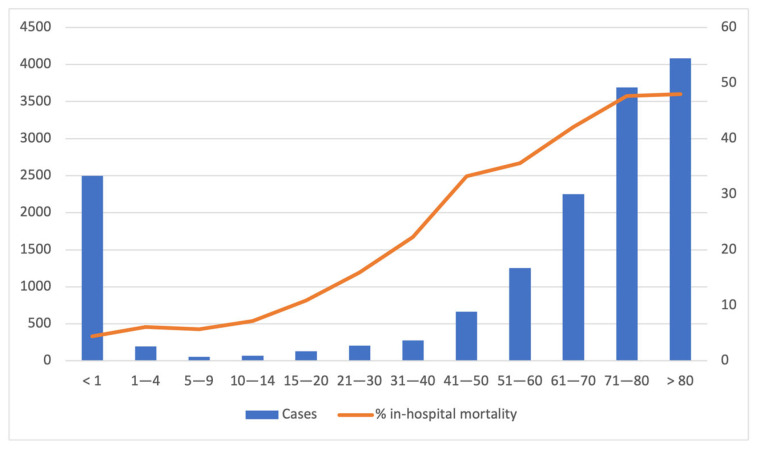
Age-specific incidence of sepsis and % in-hospital mortality from 2016 to 2020.

The average age was 58 ± 31 years, with a higher percentage of male patients (54.8%). Pediatric patients accounted for 18.5% of all hospitalizations associated with sepsis, and among these cases, 28.2% were admitted to ICUs. Specifically, there were 2495 cases of sepsis in infants (<1 year), with a mortality rate of 4.4%. This mortality rate exhibited variations, with the lowest value recorded at 3.2% in 2016 and a peak of 5.5% observed in 2019. However, the statistical analysis did not reveal a significantly different trend in mortality rates over time (*p* = 0.111). Overall, the incidence of sepsis showed a pattern of peaking in early childhood (<1 year), followed by a second peak in incidence among older adults. These trends are visually represented in Figure 3.

**Figure 4:**
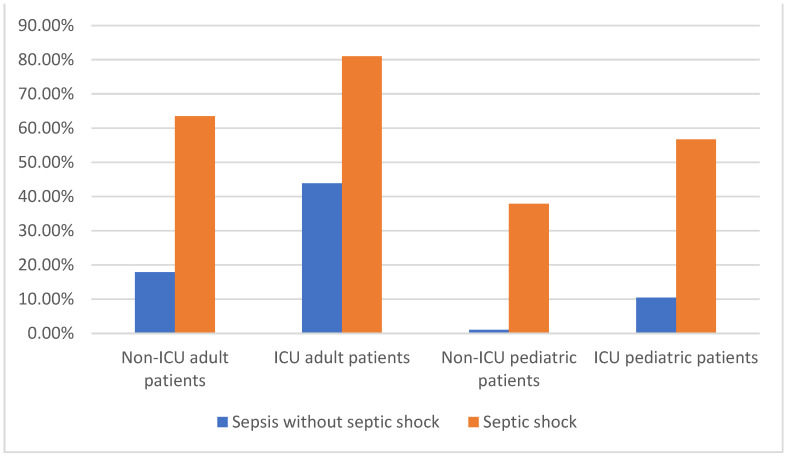
In-hospital mortality according to age (pediatric, ≤16 years) and ICU admission.

The diagnosis of septic shock was reported in 42.0% of cases. A significant increase in septic shock diagnosis was observed over the years (ß = 0.109; 95% CI: 0.03319, 0.04432; *p* < 0.001). The overall in-hospital mortality rate among patients hospitalized with sepsis was 36.3%. Specifically, in-hospital mortality rates were higher among patients with septic shock (66.7%) compared to those without septic shock (14.3%). A significant difference in in-hospital mortality was observed between adult and pediatric patients, with in-hospital mortality rates of 43.5% and 4.7%, respectively (OR = 15.68; 95% CI: 13.12–18.72; *p* < 0.001), as well as between ICU- and non-ICU-admitted patients, with in-hospital mortality rates of 52.4% and 33.5%, respectively (OR = 2.18; 95% CI: 2.00–2.39; *p* < 0.001). Among adult patients admitted to ICUs, the in-hospital mortality rate was 74.0%, whereas pediatric patients admitted to ICUs had a lower in-hospital mortality rate of 12.6%. Notably, the in-hospital mortality rate among patients transferred to ICUs after admission was 94.0%. Further stratification of this patient population according to age, ICU admission, and presence of septic shock revealed variations in in-hospital mortality rates (Figure 4). Among adult ICU-admitted patients with septic shock, the in-hospital mortality rate was highest at 81.0%, followed by non-ICU adult patients with septic shock (63.5%), ICU pediatric patients with septic shock (56.7%), ICU adult patients without septic shock (43.9%), non-ICU pediatric patients with septic shock (37.9%), non-ICU adult patients without septic shock (17.9%), ICU pediatric patients without septic shock (10.4%), and non-ICU pediatric patients without septic shock (1%).

Septic shock and older age were associated with higher in-hospital mortality rates in both adult and pediatric patients regardless of ICU admission status (Table 1).

Demographic characteristics, LOS, charges, pathogens and associated infections, and comorbidities are reported in Table 2. Sepsis was associated with Gram-positive bacteria in 11.6% of cases and with Gram-negative bacteria in 13.0% of cases. Most H-SDFs did not report the microbiological agent responsible for sepsis.

When considering the ICD-9 codes associated with septicemia, which may be indicative of sepsis associated with bacteremia, we identified a total of 9176 cases with septicemia (in-hospital mortality rate of 19.0%); in contrast, there were 6197 cases without septicemia (in-hospital mortality rate of 66.4%) (OR = 0.1; 95% CI: 0.09–0.1; *p* < 0.001).

Higher charges were observed for deceased patients (EUR 7881 ± 9335 vs. 10,548 ± 11,842; *p* < 0.001). Differences in costs were also observed between ICU and non-ICU hospitalizations (EUR 15,830 ± 11,915 vs. 7508 ± 7725; *p* < 0.001), as well as between hospitalizations for septic shock and those without septic shock (EUR 10,279 ± 9706 vs. 7629 ± 8238; *p* < 0.001). There was no significant change in charges over the study period (*p* = 0.782). The average hospital LOS was 18 ± 22 days, without significant change over the years (*p* = 0.723). A higher LOS was observed for patients admitted to ICUs in comparison to non-ICU patients (27 ± 31 vs. 17 ± 20 days; *p* < 0.001). Patients diagnosed with septic shock had a significantly longer LOS compared to those without septic shock (19 ± 22 vs. 7 ± 23 days; *p* < 0.001).

## 4. Discussion

Sepsis stands as a prominent contributor to in-hospital mortality on a global scale [3,4,5]. The utilization of hospital discharge codes derived from administrative data plays a pivotal role in elucidating the epidemiological landscape of sepsis and other medical conditions, significantly influencing epidemiological assessments. However, the true prevalence of sepsis within Italian healthcare facilities remains largely undisclosed.

In Italy, there has been a notable escalation in the number of death certificates citing sepsis, surging from 18,939 in 2003 to 49,010 in 2015, and accounting for 3% to 8% of all recorded deaths within this timeframe [17]. Additionally, recent investigations analyzing the content of death certificates in Italy have highlighted a consistent uptrend in mortality rates for the period from 2003 to 2015 [12]. We conducted an epidemiological analysis of sepsis occurrences in both ICUs and non-ICUs, revealing substantial disparities in in-hospital mortality rates, hospital lengths of stay, and associated charges. Notably, patients admitted to ICUs exhibited higher in-hospital mortality rates, prolonged hospital stays, and incurred greater financial burdens compared to those in non-ICU settings. Our findings indicated that over half of the hospitalizations for sepsis were managed outside of infectious disease units, suggesting a broader distribution of cases across various medical specialties. Furthermore, pediatric patients, defined as those under 16 years of age, accounted for 12% of all sepsis admissions, underscoring the significance of sepsis as a pediatric healthcare concern.

We identified a significant burden of hospitalizations due to sepsis and septic shock in Sicily, with the proportion of sepsis-related hospitalizations increasing compared to admissions for all causes during the study period. Additionally, the diagnoses of septic shock and in-hospital mortality rates related to sepsis also showed an upward trend over the study period.

Our analysis revealed that the overall in-hospital mortality rate among patients hospitalized with sepsis was 36.3%. This mortality rate significantly increased to 66.7% in patients diagnosed with septic shock. In contrast, patients diagnosed with sepsis without septic shock had a lower mortality rate of 14.3%. Notably, there was considerable variability in in-hospital mortality rates when considering factors such as age, admission to ICU, and the presence of septic shock. ICU-admitted patients with septic shock exhibited the highest in-hospital mortality rate (81.0%), while non-ICU-admitted pediatric patients diagnosed with sepsis without septic shock had the lowest in-hospital mortality rate (1.0%). Nevertheless, septic shock was consistently associated with elevated in-hospital mortality rates across all age groups and admission units.

An Italian study conducted in ICUs reported lower mortality rates for septic shock (69.3%) and overall sepsis without septic shock (19.2%) compared to our findings for ICU-admitted patients [14]. Similarly, a meta-analysis incorporating data from previous studies in Europe, America, and Australia estimated that the average 30-day mortality rate for septic shock was 34.7%, with a 90-day mortality rate of 38.5%. For sepsis without septic shock, the 30-day and 90-day mortality rates were even lower, at 24.4% and 32.2%, respectively [18]. These findings indicate that the mortality rates observed in our study for patients with septic shock were notably higher compared to the averages reported in the literature, whereas the mortality rates were lower for patients without septic shock. Moreover, when comparing specific subgroups according to factors such as age or ICU admission status, the disparity in mortality rates became even more pronounced for both sepsis and septic shock diagnoses. Surgery units also showed a high in-hospital mortality rate when considered alone.

It is important to acknowledge that the nature of our study does not allow us to confirm whether the diagnoses of sepsis and septic shock were accurately coded. The absence of clinical controls introduces potential biases that may impact the mortality data presented in our study. Conversely, studies in the literature that reported lower mortality rates than ours might have potentially overestimated the prevalence of sepsis by including implicit codes for sepsis in their analyses. Recent studies on sepsis epidemiology considered both explicit and implicit cases of sepsis [4,5,6,7]. Explicit cases are identified by diagnosis codes containing terms such as “sepsis”, “septicemia”, or “septic shock”, while implicit cases are defined by the simultaneous presence of codes for organ failure and infection. In our study, we chose to focus exclusively on explicit diagnosis codes to mitigate the risk of over-diagnosing sepsis. For instance, a patient initially admitted for myocardial infarction may subsequently develop cardiac heart failure, and a prolonged hospital stay may lead to hospital-acquired pneumonia. In such a scenario, the H-SDF may include codes for organ failure and infection, even though it is not a sepsis case. However, this disparity underscores the necessity for quality improvement targeting sepsis and septic shock management in Sicily, particularly for patients admitted to ICUs. While rising antibiotic resistance, particularly among patients who develop nosocomial sepsis, and an aging population with increasing comorbidities may partially account for these results, addressing issues in the diffusion of antimicrobial stewardship practices and management of septic patients, especially those progressing to septic shock, appears essential. Of note, our study revealed a concerning mortality rate of 94% among patients transferred to ICUs following their initial admission, with most cases coming from surgical units. Delayed transfers to ICUs in these cases could potentially contribute to the elevated mortality observed. However, antimicrobial stewardship programs have been implemented over the last 3 years in Sicily [19], and the expansion of intensive care capacity in response to the COVID-19 pandemic has enhanced the healthcare system’s ability to manage critically ill patients, including those with sepsis.

However, there are limited data available in the literature regarding sepsis-associated mortality. Furthermore, the literature on sepsis-associated mortality often lacks detailed stratification of patients, making direct comparisons challenging. Many studies did not provide the same level of granularity in patient categorization as our study, which complicates the interpretation and comparability of mortality rates across different groups. Furthermore, most studies analyzed only ICU-admitted patients. Collaboration to establish common frameworks for reporting and analyzing sepsis data could enhance the reliability and utility of findings.

A Chinese cross-sectional study involving patients hospitalized in ICUs revealed that approximately one-fifth of ICU admissions were attributed to sepsis, with a 90-day overall mortality rate of 35.5%. This mortality rate increased to 53.3% when considering patients with septic shock [20]. A Japanese retrospective cohort study examining adult patients admitted to ICUs with sepsis between April 2010 and March 2020 revealed higher in-hospital mortality rates for hospital-onset sepsis compared to community-onset sepsis (35.5% vs. 19.2%) [10]. It would have been interesting to conduct a similar analysis with our dataset by differentiating between nosocomial and community-acquired sepsis to assess whether the elevated mortality rates could be attributed to nosocomial infections caused by antibiotic-resistant microorganisms. Unfortunately, our H-SDFs do not allow for the distinction between in-hospital and community-acquired sepsis, limiting our ability to explore this potential association. A prospective multicenter Italian study reported an incidence of sepsis in ICUs of 11.4%, with an incidence of 32.5% for septic shock cases. Similar to our findings, the most common associated infections were pneumonia, intra-abdominal infections, and urinary tract infections [14].

Valent et al. explored potential under-coding of sepsis using ICD-9 codes. Through a comparison of H-SDFs based on ICD-9 codes with laboratory results, they found that only half of the patients with laboratory-confirmed bloodstream infections were discharged from the Italian University Hospital of Udine with a diagnosis suggestive of sepsis [15]. However, although it is known that not all cases of bacteremia are associated with sepsis, the use of ICD codes for epidemiological estimation may be subject to bias.

Agodi et al. identified *Pseudomonas aeruginosa*, *Acinetobacter baumannii*, and *Klebsiella pneumoniae* as the most frequently isolated microorganisms from sepsis episodes in ICUs [14]. Our data did not provide information about microbiological isolations and infections responsible for sepsis, except for the ICD-9 codes identifying specific septicemia and bacteremia. According to these codes, we identified a specific pathogen in one-quarter of the cases, with a slightly higher percentage for Gram-negative bacteria. Septicemia, a term previously used to describe when bacteria or other harmful microorganisms enter the bloodstream and spread throughout the body, could be considered as a surrogate marker for positive blood cultures. Our results indicate that patients with explicit septicemia codes had a significantly lower in-hospital mortality rate. Microbiological isolation is generally associated with a targeted therapy to the specific pathogen, thereby improving patient outcomes. However, therapy remains empirical in cases where the responsible microorganism is not identified and must be based on general guidelines. Another interpretation of this result, which indicates reduced in-hospital mortality among patients assigned a code indicative of septicemia, could be attributed to the historical distinction between sepsis and severe sepsis, wherein septicemia codes were primarily used to represent non-severe sepsis.

Consistent with findings reported in the literature, hospitalizations for sepsis were associated with substantial costs for the National Health System, with notably higher charges incurred for patients who died, were admitted to an ICU, or were diagnosed with septic shock. Previous studies have also demonstrated a positive association between healthcare expenditures and the severity of sepsis [16,21,22]. These findings align closely with our data, which highlight the elevated charges associated with a diagnosis of septic shock.

## 5. Conclusions

In Sicily, sepsis emerges as a significant contributor to in-hospital mortality, particularly among patients admitted to ICUs and those diagnosed with septic shock. Over the period from 2016 to 2020, there was a notable increase in the percentage of hospitalizations associated with sepsis, as well as in mortality rates associated with both sepsis and septic shock diagnoses. Hospitalizations related to sepsis incurred substantial charges, especially among patients admitted to ICUs and those diagnosed with septic shock. Patients with a septic shock diagnosis had significantly higher mortality, irrespective of age and ICU admission status. The observed mortality rates among patients with sepsis and septic shock were significantly higher than those reported in previously published studies, particularly among ICU-admitted patients. The retrospective nature of our study and the lack of control over the validity of the assignment of sepsis diagnostic codes may have affected our results. In any case, these findings demonstrate the need for careful monitoring of the causes of death of patients hospitalized in intensive care in Sicily; of the application of antimicrobial stewardship, as well as infection prevention and control programs; and of the correct coding of H-SDFs.

## 6. Limitations

This study faced several limitations that warrant consideration. Firstly, the identification of sepsis using the ICD-9 CM code algorithm might lack precision compared to screening based on clinical criteria, potentially resulting in over-coding or under-coding. Secondly, the retrospective nature of this study precluded the consideration of confounding factors that may have resulted in higher mortality rates, such as the accuracy of code assignments. Lastly, our study lacked comprehensive blood exams and complete microbiological data, except for some information reported in the H-SDFs.

## Figures and Tables

**Table 1:** Sepsis and septic shock frequencies according to groups.

Variables	All Hospitalization	Vital Status at Discharge		OR (95% CI)	*p*
		Alive	Dead		
Adult Non-ICU Hospitalizations					
	N = 11,030	N = 6690	N = 4340		
Average age ± standard deviation (years)	72 ± 15	70 ± 16	75 ± 12		<0.001
Sepsis without septic shock	5846 (53.0%)	(71.6%)	1048 (24.1%)		
Septic shock	5184 (47.0%)	1892 (28.4%)	3292 (75.9%)	7.966 (7.298, 8.695)	<0.001
Adult ICU hospitalizations					
	N = 1478	N = 384	N = 1094		
Average age ± standard deviation (years)	68 ± 14	64 ± 16	69 ± 13		<0.001
Sepsis without septic shock	278 (18.8%)	(40.6%)	122 (11.2%)		
Septic shock	1200 (81.2%)	228 (59.4%)	972 (88.8%)	5.451 (4.131, 7.193)	<0.001
Pediatric non-ICU hospitalizations					
	N = 2048	N = 2016	N = 32		
Average age ± standard deviation (years)	1 ± 3	1 ± 3	3 ± 6		<0.001
Sepsis without septic shock	2019 (98.6%)	(99.1%)	21 (65.6%)		
Septic shock	29 (1.4%)	18 (0.9%)	11 (34.4%)	58.143 (24.492, 138.031)	<0.001
Pediatric ICU hospitalizations					
	N = 804	N = 703	N = 101		
Average age ± standard deviation (years)	0 ± 2	0 ± 2	1 ± 2		0.028
Sepsis without septic shock	767 (95.4%)	(97.7%)	80 (79.2%)		
Septic shock	37 (4.6%)	16 (2.3%)	21 (20.8%)	11.271 (5.651, 22.480)	<0.001

**Table 2:** Description of demographic features, LOS, pathogens and associated infections, and comorbidities of patients hospitalized for sepsis in Sicily according to vital status at discharge.

		Vital Status at Discharge	
Variables	All Hospitalization N = 15,373	Alive N = 9793	Dead N = 5580
Sex			
Male	8396 (54.8%)	5456 (55.9%)	2940 (52.7%)
Female	6934 (44.3%)	4298 (44.1%)	2636 (47.3%)
Age (average ± SD)	58 ± 31	50 ± 34	72 ± 17
Average LOS	18 ± 22	18 ± 21	17 ± 23
Average charges	8849 ± 10,395	7881 ± 9335	10,548 ± 11,842
Septic shock	6462 (42.0%)	2154 (22.0%)	4308 (77.2%)
Septicemia codes	9176 (59.7%)	7710 (78.7%)	1466 (26.3%)
Gram-positive sepsis	1787 (11.6%)	1540 (15.7%)	247 (4.4%)
Gram-negative sepsis	1999 (13.0%)	1665 (17.0%)	334 (6.0%)
Non-specified pathogen sepsis	11,643 (75.7%)	6631 (80.3%)	5012 (94.0%)
Adult non-ICU	11,043 (71.8%)	6690 (68.3%)	4353 (78.0%)
Adult ICU	1478 (9.6%)	384 (3.9%)	1094 (19.6%)
Pediatric non-ICU	2048 (13.3%)	2016 (20.6%)	32 (0.6%)
Pediatric ICU	804 (5.2%)	703 (7.2%)	101 (1.8%)
Associated pathogens (%)			
Human immunodeficiency virus	72 (0.5%)	54 (0.6%)	18 (0.3%)
Hepatitis C virus	78 (0.5%)	60 (0.6%)	18 (0.3%)
Tuberculosis	90 (0.6%)	66 (0.7%)	24 (0.4%)
*Streptococcus* species	242 (1.6%)	216 (2.2%)	26 (0.5%)
*Staphylococcus* species	1749 (11.4%)	1452 (14.8%)	297 (5.3%)
*Staphylococcus aureus*	712 (4.6%)	594 (6.1%)	118 (2.1%)
*Klebsiella pneumoniae*	174 (1.1%)	64 (0.7%)	110 (2.0%)
*Pseudomonas aeruginosa*	402 (2.6%)	292 (3.0%)	110 (2.0%)
*Escherichia coli*	910 (5.9%)	808 (8.3%)	102 (1.8%)
*Clostridioides difficile*	66 (0.4%)	32 (0.3%)	34 (0.6%)
*Candida* species	245 (1.6%)	168 (1.7%)	77 (1.4%)
*Aspergillus* species	14 (0.1%)	4 (<0.1%)	10 (0.1%)
Associated infection (%)			
Pneumonia	2499 (16.3%)	1251 (12.8%)	1248 (22.4%)
Endocarditis	165 (1.1%)	124 (2.3%)	41 (0.7%)
Osteomyelitis	90 (0.6%)	80 (0.8%)	10 (0.2%)
Pyelonephritis	170 (1.1%)	148 (1.5%)	22 (0.4%)
Urinary tract infection	836 (5.4%)	670 (6.8%)	166 (3.0%)
Meningitis	61 (0.4%)	44 (0.4%)	61 (0.4%)
Biliary tract infection	522 (3.4%)	338 (3.5%)	184 (3.3%)
Gastrointestinal infection	222 (1.4%)	150 (1.5%)	72 (1.3%)
Peritonitis	540 (3.5%)	150 (1.5%)	390 (7.0%)
Appendicitis	25 (0.2%)	19 (0.2%)	6 (0.1%)
Intrabdominal abscesses	231 (1.5%)	146 (1.5%)	85 (1.5%)
Skin and skin structure infection	92 (0.6%)	72 (0.7%)	20 (0.4%)
Comorbidities (%)			
Hematological malignancy	657 (4.3%)	471 (4.8%)	186 (3.3%)
Malignant tumor	1122 (7.3%)	675 (6.9%)	447 (8.0%)
Dementia	335 (2.2%)	183 (1.9%)	152 (2.7%)
Alcoholism	83 (0.5%)	38 (0.4%)	45 (0.8%)
Obesity	153 (1.0%)	67 (0.7%)	86 (1.5%)
Chronic cardiac failure	178 (1.2%)	100 (1.0%)	78 (1.4%)
Chronic cardiac ischemia	559 (3.6%)	297 (3.0%)	262 (4.7%)
Hypertension	752 (4.9%)	530 (5.4%)	222 (4.0%)
Chronic renal failure	1364 (8.9%)	842 (8.6%)	522 (9.4%)
Dialysis	212 (1.4%)	145 (1.5%)	67 (1.2%)
Diabetes	1528 (9.9%)	1004 (10.3%)	524 (9.4%)
Chronic obstructive pulmonary disease	544 (3.6%)	306 (3.1%)	238 (4.3%)
Chronic respiratory failure	764 (5.0%)	314 (3.2%)	450 (8.1%)
Non-alcoholic hepatic cirrhosis	315 (2.0%)	213 (2.2%)	102 (1.8%)
Alcoholic hepatic cirrhosis	70 (0.5%)	37 (0.4%)	33 (0.6%)
Intestinal bowel disease	49 (0.3%)	25 (0.3%)	24 (0.4%)

## Data Availability

Data are available upon request to corresponding author.
